# Associations of Lower Limb Muscle–Tendon Properties with Dual-Task Gait Variability: A Cross-Age Study

**DOI:** 10.3390/healthcare13121375

**Published:** 2025-06-09

**Authors:** Zheng Dong, YoungJin Moon, Sang Ki Lee, Hwi-yeol Yun, JuWon Song, JiaHao Xu, Min Ju Shin, DuBin Im, XuanRu Wang

**Affiliations:** 1 Department of Sport Science, Chungnam National University, Daejeon 34134, Republic of Korea; dongzheng622@naver.com (Z.D.);; 2 College of Pharmacy, Chungnam National University, Daejeon 34134, Republic of Korea

**Keywords:** gait variability, muscle–tendon stiffness, MyotonPRO, aging, dual task

## Abstract

Objectives: This study is the first to investigate the association between lower limb muscle–tendon mechanical properties and dual-task gait variability using a handheld, non-invasive myotonometer (MyotonPRO). Methods: A cross-sectional design was employed, involving 48 participants (older adults: 72.05 ± 3.52 years; younger adults: 24.8 ± 2.36 years). The stiffness and elasticity of dominant lower limb muscles and tendons were assessed using the MyotonPRO. Gait variability—including step length, stride length, and gait cycle time—was measured using the OptoGait system. Results: Compared to the younger group, older adults showed increased stiffness of the patellar tendon (*p* < 0.001) and decreased stiffness of the Achilles tendon (*p* < 0.047). Additionally, both the rectus femoris and biceps femoris exhibited significantly higher stiffness (*p* < 0.05) and reduced elasticity (*p* < 0.001). Patellar tendon stiffness was positively correlated with gait variability (r = 0.55 to 0.68, *p* < 0.01), whereas Achilles tendon stiffness showed a negative correlation (r = −0.32 to −0.40, *p* < 0.05). Conclusions: This study provides preliminary evidence linking muscle–tendon mechanical properties with dual-task gait stability in older adults. Increased stiffness in the patellar tendon and decreased stiffness in the Achilles tendon suggest these structural characteristics may play a crucial role in gait control and hold potential as predictive markers of fall risk. Linking non-invasive MyotonPRO-derived mechanical properties with key spatiotemporal gait parameters may support its potential use in the early detection of gait instability in older adults.

## 1. Introduction

Falls are a major health concern among older adults aged 65 years and above, with mortality and disability rates increasing significantly with age [1]. Studies have shown that abnormalities in gait characteristics are closely associated with fall risk in the elderly, and changes in spatiotemporal gait parameters are often regarded as early indicators [2]. Standardized investigations on these parameters have been conducted in older populations, providing important references for clinical assessments [3]. Previous studies have reported that older adults and individuals with cognitive impairments often exhibit greater gait variability [4,5]. This increased variability reflects a decline in the automatic regulation of walking and is associated with a heightened risk of falling in these populations [3]. Several studies have confirmed that specific gait variability metrics—such as step length, stride length, and gait cycle time variability—are linked to fall history [6], frailty [7], muscle strength, and balance function [5,8]. Among these, stride length variability has been identified as a reliable predictor distinguishing fallers from non-fallers [9]. Based on previous findings, gait variability has been increasingly regarded as a potential indicator of walking stability and fall risk [10,11,12].

The dual-task paradigm, widely used to evaluate gait control ability, has shown particular advantages in exploring cognitive–motor interaction mechanisms. Evidence indicates that the introduction of cognitive load (e.g., serial subtraction tasks) significantly affects gait performance [13], with these effects being particularly pronounced in older adults at high risk of falling [14]. It is worth noting that healthy older adults often adopt more conservative gait strategies under dual-task conditions, such as reduced walking speed and shortened stride length [15]. These adjustments may not necessarily indicate decreased stability but rather reflect age-related adaptive protective mechanisms.

Degenerative changes in the neuromuscular system are the physiological basis for increased gait variability. Age-related declines in musculoskeletal and neural function significantly impair motor control, resulting in decreased gait quality and increased variability [5,16]. Furthermore, abnormal increases in gait variability may not only indicate physiological aging but also suggest underlying neurodegenerative diseases or musculoskeletal impairments.

Importantly, the mechanical properties of muscle–tendon structures play a critical role in maintaining gait stability by regulating force transmission and energy efficiency during movement [17,18]. However, these biomechanical properties undergo significant age-related alterations, including increased stiffness, fat infiltration, changes in muscle fiber type, and abnormal collagen metabolism [19,20]. Current evidence suggests that age-related changes in muscle–tendon stiffness [21,22] may lead to reduced force transmission efficiency, compromised gait efficiency, and impaired dynamic balance, thereby elevating the risk of falls.

Shear wave elastography (SWE) has recently been employed for the quantitative evaluation of muscle–tendon stiffness, demonstrating unique value in predicting physical frailty [23] and monitoring age-related musculoskeletal changes [24]. This technique estimates Young’s modulus by measuring shear wave propagation speed, providing both visual and quantitative insights. However, its high cost, technical complexity, and operator dependence limit its widespread clinical application. Although the development of portable SWE devices [25] has alleviated some operational challenges, issues such as measurement reproducibility remain.

In contrast, the MyotonPRO—a portable, user-friendly, and cost-effective non-invasive device—has attracted increasing attention in the fields of orthopedics and sports medicine. It is widely used by clinicians and physical therapists to assess muscle function and soft tissue status [26], and shows promise for identifying age-related functional decline [27] and stratifying fall risk [28]. The device provides rapid assessments of mechanical parameters such as muscle tone, stiffness, and elasticity. Its ease of use and high repeatability make it particularly suitable for bedside and rehabilitation settings, comparable to accelerometers in terms of speed and practicality. Notably, accelerometers have been successfully applied in clinical studies of gait variability, particularly in evaluating postoperative functional recovery following knee arthroplasty [29] and tibial plateau fracture surgery [30]. Despite these advantages, no studies to date have explored the relationship between lower limb muscle–tendon mechanical properties and gait variability under dual-task conditions using the MyotonPRO.

Given this research gap, the present study aimed to (1) compare lower limb muscle–tendon stiffness and elasticity between older and younger adults; (2) investigate the association between these biomechanical parameters and gait variability (including step length, stride length, and gait cycle time variability) under dual-task conditions; (3) explore the feasibility of using a handheld, non-invasive device (MyotonPRO) as an indirect tool for assessing gait variability.

## 2. Materials and Methods

### 2.1. Participants

Young participants were recruited from a university setting, while older participants were recruited from local senior activity centers. All participants included in this study were right-leg dominant. In cases where dominance was unclear, participants were asked which leg they would use to kick a ball in order to determine leg dominance. All participants were required to have sufficient cognitive ability to complete the study tasks. Exclusion criteria included a history of lower limb fracture or injury at the test site, presence of neurological or musculoskeletal disorders, diagnosis of connective tissue diseases (e.g., scleroderma, lupus, or polymyositis/dermatomyositis), or physical frailty that prevented completion of treadmill walking tasks. The study was approved by the Institutional Review Board of the university (IRB: 202405-SB-059-01), and all participants provided written informed consent prior to participation.

### 2.2. Sample Size and Power Analysis

An a priori power analysis for correlation was performed using G*Power 3.1 (Heinrich Heine University Düsseldorf, Düsseldorf, Germany) to determine the required sample size. Given the exploratory nature of the study, an effect size of r = 0.40 (medium to large), α = 0.05, and a desired power of 0.80 were used. The analysis indicated that a minimum of 44 participants was required. To account for potential dropouts, data loss, and the relatively intensive measurement protocol, 60 participants (30 older adults and 30 younger adults) were initially recruited. However, due to participant withdrawal and data exclusions related to technical errors, the final analysis included 48 participants: 26 younger adults (13 males, 13 females) and 22 older adults (8 males, 14 females). Although slightly below the ideal estimate, the final sample met the minimum requirement and yielded statistically significant results.

### 2.3. Stiffness and Elasticity Measurements

**Figure 1:**
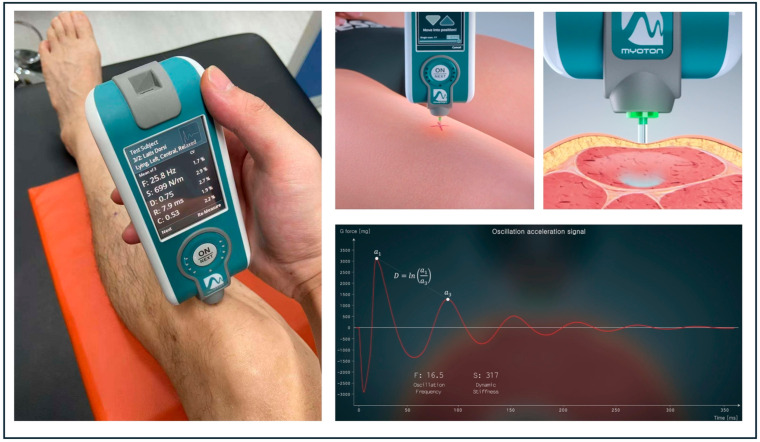
The left panel shows the on-site measurement of the patellar tendon using the MyotonPRO device. The right images depict the official schematic representation of MyotonPRO’s measurement principle, demonstrating the application of mechanical impulses and tissue response (image from myoton.com. Accessed on 17 April 2025).

A handheld, non-invasive device called the MyotonPRO (Myoton AS, Tallinn, Estonia) was used to assess muscle stiffness. The MyotonPRO is a reliable and widely used tool for evaluating muscle stiffness and elasticity, and is considered an effective method for assessing the mechanical properties of muscle tissue. Elasticity (D, dimensionless; logarithmic ratio) reflects the damping characteristics of the tissue and is calculated as D = $Ln\left(\frac{a_{1}}{a_{3}}\right)$, representing the rate at which oscillations decay after mechanical perturbation. Importantly, D values are inversely related to tissue elasticity: a lower D value indicates longer-lasting oscillations and better recovery capacity, corresponding to higher elasticity. Conversely, a higher D value reflects faster damping and reduced elasticity, typically observed in stiffer tissues. Stiffness (S, N/m) reflects the biomechanical resistance of the muscle to deformation and is calculated using the formula S = $\frac{a_{max\cdot }m_{probe}}{∆l}$. Measurement regions included thigh muscles (rectus femoris and biceps femoris), lower leg muscles (tibialis anterior and soleus), and tendinous structures (Achilles and patellar tendon). All measurements were conducted by trained researchers strictly following the official MyotonPRO user manual (Figure 1). Participants rested in a supine or prone position for 10 min before each measurement to ensure complete muscle relaxation. The measurement locations for muscles and tendons were determined based on the methodology described in previous studies [31], in combination with the standardized measurement guidelines provided on the official MyotonPRO website.

Each measurement point was assessed three times, with a 15 s interval between trials. If the coefficient of variation (CV) exceeded 3%, the measurement was repeated until the CV fell below this threshold. The average of the three trials was used for subsequent analysis.

### 2.4. Gait Measurement

**Figure 2:**
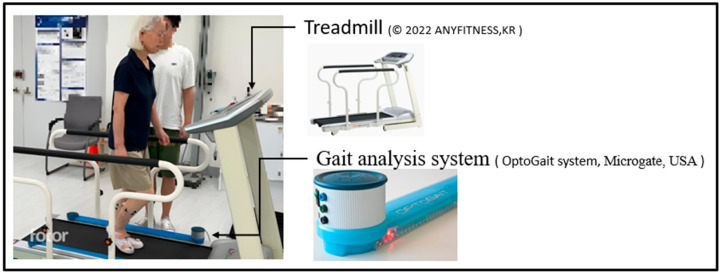
Treadmill gait assessment with the OptoGait system.

Prior to testing, participants wore properly fitted, laboratory-provided footwear and were familiarized with the walking tasks. A warm-up trial was conducted on the treadmill, during which an experienced researcher helped determine two walking speeds: self-selected and fast-paced. The self-selected speed represented the participant’s usual, comfortable walking pace, whereas the fast-paced speed was brisk yet sustainable. Some older participants reported being unfamiliar with treadmill walking. To address this, adaptation training was provided on both level ground and the treadmill to minimize measurement error caused by inexperience (Figure 2). The adaptation protocol was as follows: (1) Participants first dismounted the treadmill and walked freely on level ground. The researcher explained the basic skills of treadmill walking and guided the participants to practice multiple times on the ground to encourage their natural walking pattern. (2) After sufficient ground practice, participants returned to the treadmill for walking practice, during which the researcher provided further instruction on proper treadmill walking techniques (e.g., increasing stride length as speed increases, walking forward rather than marching in place). (3) Once participants reported being able to complete both self-selected and fast-paced walking conditions comfortably, and the researcher deemed their gait to be stable, the adaptation phase was considered complete.

Prior to formal testing, participants completed four treadmill walking tasks at two speeds: (1) self-selected speed, normal condition; (2) self-selected speed, dual-task condition (serial subtraction of 7); (3) fast-paced speed, normal condition; (4) fast-paced speed, dual-task condition (serial subtraction of 7). During dual-task conditions, the researcher randomly selected a starting number below 200, from which participants continuously subtracted 7. If an error occurred, the researcher immediately corrected it and instructed the participant to resume from the corrected number until the walking trial was completed. Short breaks were provided between tasks to ensure participants were fully rested before proceeding. Once readiness was confirmed, the next task began. Each walking task lasted 4 min, with data analysis focused on the middle 2 min to capture steady-state gait.

Gait parameters were measured using the OptoGait system (Microgate, Mahopac, NY, USA), which recorded stride length, step length, gait cycle time, and the coefficient of variation (CV) for each parameter. Gait variability was automatically calculated using the following formula: CV (%) = 100 × (standard deviation/mean). In this study, the CV of step length, stride length, and gait cycle time were used as key indicators of gait variability.

### 2.5. Statistical Analysis

Data normality was assessed using the Shapiro–Wilk test, supplemented by evaluations of skewness and kurtosis. Independent sample t-tests were used to compare baseline characteristics between groups. Descriptive statistics are reported as mean ± standard deviation (M ± SD).

Spearman’s rank-order correlation was performed to assess the association between gait variability parameters and the mechanical properties of the right lower limb. Correlation coefficients (r) were interpreted as indicators of effect size, with values of approximately 0.10, 0.30, and 0.50 representing small, moderate, and large effects, respectively, based on Cohen’s guidelines. Multiple linear regression analyses were additionally conducted to control for the potential confounding effect of age. The Wilcoxon signed-rank test was used to examine within-group differences in stiffness and elasticity, whereas the Mann–Whitney U test was applied for between-group comparisons.

A two-way repeated-measures multivariate analysis of variance (MANOVA) with a 2 × 4 (group × condition) design was conducted to compare gait performance variables, including step length variability, stride length variability, and gait cycle time variability. When appropriate, Bonferroni correction was applied for post hoc comparisons.

Statistical significance was set at *p* < 0.05. All statistical analyses were performed using IBM SPSS Statistics (version 29.0; IBM Corp., Armonk, NY, USA).

## 3. Results

### 3.1. Subject Characteristics

Participant characteristics are presented in Table 1. No significant between-group difference was observed in baseline body weight; however, significant differences were found in age, height, BMI, walking speed, and step length.

### 3.2. Gait Variability

**Figure 3:**
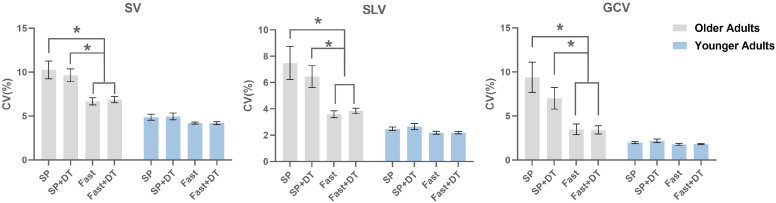
Gait variability under four walking conditions in older and younger adults. SV = step length variability; SLV = stride length variability; GCV = gait cycle time variability; SP = self-paced walking; DT = dual task. Error bars represent the standard error of the mean (SEM). Significant differences between conditions are indicated by * *p* < 0.001.

The MANOVA revealed significant main effects of group and task, and a group × task interaction for all gait variability parameters (SV, SLV, GCV; all *p* < 0.001; Table 2). In younger adults, gait variability remained relatively stable across all walking conditions. In contrast, older adults demonstrated significantly reduced SV, SLV, and GCV in the SP + DT, Fast, and Fast + DT conditions compared to the SP condition (all *p* < 0.001; Figure 3).

### 3.3. Mechanical Properties of Muscles and Tendons

**Figure 4:**
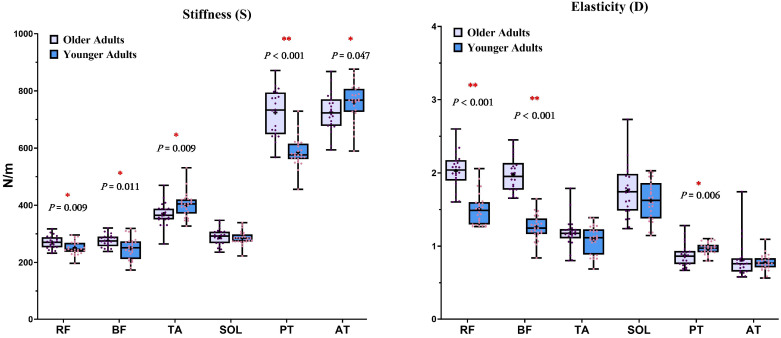
Scatter plot for the groups’ right leg muscles and tendons mechanical properties. RF = rectus femoris; BF = biceps femoris; TA = tibialis anterior; SOL = soleus; PT = patellar tendon; AT = Achilles tendon. Each dot represents an individual value; horizontal bars indicate median values. Significant group differences are indicated as follows: * *p* < 0.05; ** *p* < 0.001.

Table 3 presents the differences in stiffness and elasticity parameters for various muscles and tendons in both legs between the two groups. Figure 4 displays scatter plots illustrating the range of stiffness (S) and elasticity (D) for various muscles and tendons in both groups.

As shown in Figure 4, older adults exhibited significantly higher S values in the rectus femoris (RF), biceps femoris (BF), and patellar tendon (PT) compared to younger adults (*p* < 0.05). In contrast, the S values of the tibialis anterior (TA; *p* = 0.009) and Achilles tendon (AT; *p* = 0.047) were significantly lower in the older group.

Additionally, older adults showed significantly higher D values for the RF and BF (*p* < 0.001), but significantly lower D values in the PT (*p* = 0.006), relative to younger adults. These results, obtained using a portable device, provide objective insights into age-related changes in muscle and tendon mechanical properties.

Table 4 shows that PT S values were positively associated with all gait variability indices (r = 0.55 to 0.68, *p* < 0.01), while AT S values showed negative correlations with SV, SLV, and GCV (r = −0.32 to −0.40, *p* < 0.05). RF S values were also positively correlated with SV and GCV under both SP and Fast + DT conditions (r = 0.30 to 0.37, *p* < 0.05). Both BF and RF D values were positively associated with SV, SLV, and GCV across all conditions (r = 0.49 to 0.69, *p* < 0.01). In contrast, PT D values were negatively correlated with gait variability (r = −0.30 to −0.39, *p* < 0.05).

As shown in Appendix A Table A1, under the SP condition, PT_S, RF_D, and BF_D significantly and positively predicted all three gait variability metrics (*β* = 0.42 to 0.61, *p* < 0.05). However, after including age as a covariate, these positive associations were generally attenuated. Some *p*-values increased to > 0.10, and in certain cases, the direction of the regression coefficients even reversed. The significance levels of ΔF(+Age) suggest that these associations may be driven by the confounding effect of age.

**Figure 5:**
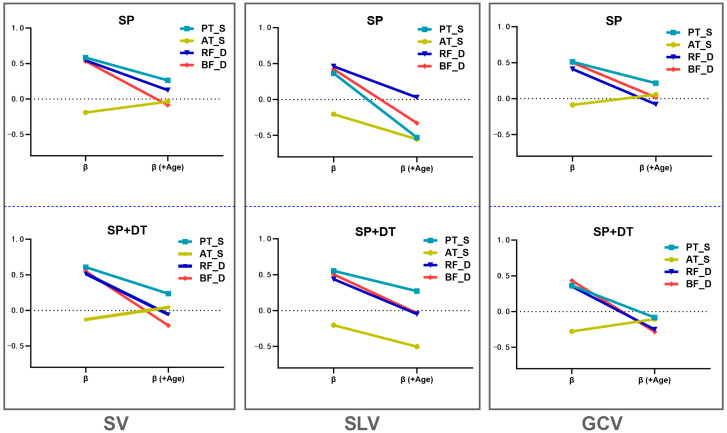
Changes in standardized regression coefficients (β) of tendon and muscle properties before and after adjusting for age. Multiple linear regression analyses were conducted under both single-task and dual-task self-selected speed conditions (SP and SP + DT), examining three gait variability parameters—step length variability (SV), stride length variability (SLV), and gait cycle variability (GCV). These analyses assessed how the standardized regression coefficients (β) of tendon/muscle variables changed before and after controlling for age. The lines in the figure represent patellar tendon stiffness (PT_S), Achilles tendon stiffness (AT_S), rectus femoris elasticity (RF_D), and biceps femoris elasticity (BF_D). The results highlight the confounding role of age in the observed associations.

Moreover, as illustrated in Figure 5, under the SV-SP condition, AT_S initially exhibited a negative regression coefficient (*β* = −0.206), which approached zero after adjusting for age—indicating a lack of independent predictive power, with its effect potentially mediated by age. However, under the SLV condition, the negative predictive effect of AT_S actually strengthened after age adjustment, suggesting that age may have masked a true underlying relationship, i.e., greater stiffness was associated with lower stride length variability. By contrast, for SV and GCV, the negative association between AT_S and gait variability essentially disappeared after controlling for age (*β* approaching 0), indicating parameter-specific effects.

Further analysis under the dual-task (SP + DT) condition revealed a 10–20% additional decrease in the β coefficients for the aforementioned positive predictors. Both ΔR² and ΔF(+Age) values were generally higher than those under the single-task condition, suggesting that cognitive load may amplify the confounding effect of age. Overall, Figure 5 and Appendix A Table A1 visually underscore the crucial role of age in explaining the relationships between tendon/muscle mechanical properties and gait variability.

**Figure 6:**
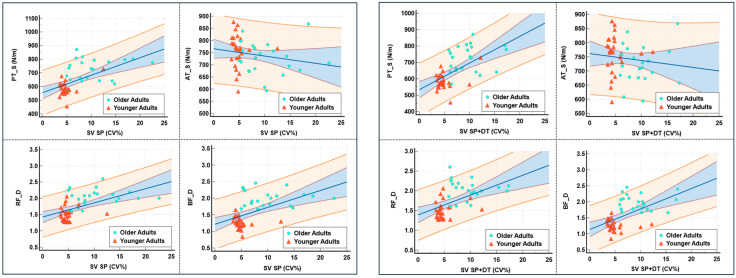
Regression scatter plots of step length variability under dual-task walking and tendon/muscle mechanical properties. The scatter plots illustrate the associations between step length variability (SV, CV%) and four mechanical parameters—patellar tendon stiffness (PT_S), Achilles tendon stiffness (AT_S), rectus femoris elasticity (RF_D), and biceps femoris elasticity (BF_D) during both single-task (SP) and dual-task self-selected speed (SP + DT) walking. Participants are color-coded by age group: younger adults (red triangles) and older adults (green circles). The blue solid line indicates the overall regression trend with 95% confidence intervals (blue shaded area), while the orange dashed bands represent group-specific confidence intervals.

To further illustrate the moderating effect of age, regression scatter plots were generated to depict the associations between step length variability (CV, %) and the four tendon/muscle variables under both single-task and dual-task conditions (Figure 6). The results showed that under the SP condition, CV was positively associated with PT_S, RF_D, and BF_D. These associations appeared to be largely driven by the older adult group. As CV (%) increased, the confidence intervals of the fitted lines widened considerably, indicating greater prediction uncertainty with higher variability.

## 4. Discussion

This cross-age comparative study is the first to systematically examine the relationship between lower limb muscle–tendon mechanical properties, as measured by a handheld myotonometer (MyotonPRO), and gait variability—addressing a previously underexplored gap in the literature. The results revealed that age significantly moderates the association between muscle–tendon mechanical properties and gait control. This moderating effect appears to be further amplified under dual-task conditions. Increased stiffness of the patellar tendon, decreased stiffness of the Achilles tendon, and reduced elasticity (i.e., higher D values) of the rectus femoris and biceps femoris emerged as prominent structural correlates in the older adult group, showing significant associations with gait variability.

Under the dual-task self-selected speed condition (SP + DT), older adults exhibited reduced gait variability (SV, SLV, and GCV), contrasting with prior studies that reported increased gait variability under dual-tasking [32]. According to the “posture-first” strategy, older adults may prioritize postural control over cognitive task performance when faced with dual-task demands. In this context, increased cognitive load may prompt a shift of attentional resources toward gait stability, thereby reducing gait variability as a compensatory mechanism [33]. However, this interpretation remains speculative and requires further investigation. Moreover, a decrease in gait variability does not necessarily indicate improved stability—it may instead reflect compensatory strategies [15]. The observed gait slowing in older adults during dual-task walking could be an adaptive “cautious gait” aimed at minimizing fall risk by reducing motor complexity [30]. It is noteworthy that older adults demonstrated higher gait variability at self-selected speeds (SP) but reduced variability at faster walking speeds (Fast). One possible explanation is that slower walking may limit the activation of automatic gait regulation mechanisms. Walking speed itself, as a mediating variable, is closely tied to age-related changes in spatiotemporal gait parameters [34]. It is also important to consider differences between treadmill and overground walking—treadmill rhythmicity may alter gait adaptation strategies [35], such as kinematic compensation to match the belt motion. Notably, under near-maximal conditions such as fast dual-task walking (Fast + DT), the added cognitive demand may exceed older adults’ cognitive–motor integration capacity, resulting in gait deterioration [4,15,29]. These findings further support the view that gait control requires continuous cognitive engagement, particularly in aging populations, and suggest a speed-dependent “strategy switching” mechanism during dual-task gait in older adults. This finding is consistent with previous reports. Older adults typically show slower gait speed and shorter stride length [32,36,37,38]. Nonetheless, it should be noted that the treadmill-based walking environment and the specific dual-task paradigm employed in this study may have influenced participants’ attentional resource allocation. These methodological factors could limit the ecological validity of the findings; therefore, caution is warranted when generalizing the results to real-world gait scenarios.

Importantly, this study found that reduced elasticity in the rectus femoris and biceps femoris was associated with increased gait variability, supporting the theoretical framework that muscle–tendon mechanical properties are key determinants of dynamic balance and gait stability [19,39]. However, when age was included as a covariate in the regression models, the strength of these associations was markedly attenuated, suggesting that aging itself may be a primary driver of the link between muscle mechanical properties and gait variability. Previous studies have indicated that age-related increases in muscle stiffness may impair the body’s ability to absorb and adapt to gait perturbations, thereby increasing fall risk [40,41].

This study also observed significant differences in patellar tendon mechanical properties between older and younger adults, potentially related to the accumulation of non-enzymatic cross-links such as advanced glycation end-products (AGEs), which can increase stiffness and reduce elasticity [42,43]. Although the association between patellar tendon stiffness and gait variability was attenuated after adjusting for age, Figure 6 still revealed a positive linear trend in the older group. This suggests that the underlying mechanisms may warrant further investigation. However, due to the limited sample size, these observed age-related adaptations may not be generalizable to broader populations, and caution is warranted when interpreting their clinical relevance. It is worth noting that moderate tendon stiffness may enhance gait performance by improving force transmission efficiency [23,44], whereas age-related neuromuscular declines (e.g., reduced plantarflexor propulsion) may compel older adults to adopt atypical gait patterns [30]. Moreover, older adults often retain greater eccentric than concentric strength, potentially due to structural stiffening that alters motor control strategies [20]. Hence, the mechanical properties of the calf–Achilles tendon complex likely play a critical role in gait stability. The observed negative correlation between Achilles tendon stiffness and gait variability (i.e., greater stiffness associated with lower variability) should be interpreted with caution, as it may be confounded by age. Additionally, discrepancies between treadmill and overground walking conditions may further limit the generalizability of these mechanical–functional associations to real-world gait performance. Nevertheless, targeted interventions aimed at improving Achilles tendon stiffness could still prove beneficial in enhancing older adults’ ability to respond to sudden gait disturbances [45], warranting further investigation. Recent systematic reviews lend further support to these findings. A meta-analysis by Delabastita et al. highlighted that decreased Achilles tendon stiffness in older adults is closely linked to impaired walking ability and balance [46]. Boyer et al. (2023) further emphasized that age-related changes in tendon–muscle unit mechanics are a key contributor to increased metabolic cost during walking in older adults [47]. The “Achilles tendon compliance–energy cost” hypothesis also suggests that reduced stiffness disrupts neuromechanical synergy in the calf muscles, exacerbating gait instability and energy inefficiency [48].

This study identified statistically significant associations between several tendon–muscle mechanical properties and gait variability across different walking conditions, based on a series of regression and correlation analyses. Importantly, all hypothesis tests were guided by theory-driven models (e.g., the link between Achilles tendon stiffness and stride length variability), rather than post hoc data mining. While multiple comparison corrections may mask weak but biologically relevant effects, the focus on consistent, biomechanically plausible variables across multiple conditions minimized the risk of overinterpretation. Nonetheless, caution is warranted when interpreting marginally significant associations due to the risk of false positives. These findings should be regarded as preliminary and hypothesis-generating, warranting replication in larger and longitudinal cohorts.

The findings of this study suggest that non-invasive, handheld myotonometers such as the MyotonPRO hold significant promise for clinical application in geriatric care. With advantages including ease of use, rapid measurement, and high repeatability, these devices are particularly suitable for screening and assessment in frail older adults. Their portability facilitates integration into primary care settings, community-based fall prevention programs, and bedside evaluations in long-term care facilities, as part of comprehensive geriatric assessments. Increased stiffness of the patellar tendon and decreased stiffness of the Achilles tendon—identified in this study—were found to be associated with greater gait variability and may represent potential surrogate indicators. Compared to conventional gait analysis systems, handheld devices provide a more convenient, indirect means of gait assessment without requiring actual walking tasks. Future research using prospective designs and receiver operating characteristic (ROC) curve analysis could help establish clinically meaningful thresholds for predicting fall risk. Once operational cut-off values are validated, these tendon–muscle mechanical properties may be used to identify individuals at high risk of falling and serve as practical tools for personalized fall risk management and early intervention.

Several limitations should be acknowledged. First, the cross-sectional design precludes causal inference, and potential confounding effects from long-term physical activity habits or chronic conditions cannot be ruled out. Future studies should employ longitudinal designs and include lifestyle covariates to strengthen causal interpretations. Second, although cross-age comparisons revealed general trends, the relatively small sample size (n = 48) may limit statistical power, and larger-scale studies are needed for more robust conclusions. Third, the MyotonPRO device is limited to assessing superficial tissue properties, potentially introducing measurement bias for deeper muscles/tendons. Future research should consider combining MyotonPRO with ultrasound elastography for more comprehensive assessments. Fourth, sex differences were not analyzed in this study, despite prior evidence indicating that mechanical properties such as tendon stiffness are influenced by sex (e.g., males typically exhibit greater stiffness and strength) [22,31,49]. Future studies should include balanced samples and subgroup analyses to explore the moderating effect of sex. Lastly, treadmill walking in a laboratory setting differs from overground walking in ecological validity. Although previous studies report no significant differences in spatiotemporal means [50], treadmill-induced rhythmicity and altered optical flow may affect gait patterns [34,51]. Despite the use of an adaptation protocol in this study, individual differences in treadmill acclimation—particularly among older adults—may have influenced the results. Future studies should aim to validate these findings in more naturalistic walking environments to enhance ecological validity and generalizability.

## 5. Conclusions

This study identified a significant association between the stiffness and elasticity of lower limb muscles and tendons—measured using a non-invasive handheld device—and gait variability. Compared to younger adults, older adults exhibited greater stiffness in the rectus femoris, biceps femoris, and patellar tendon, along with reduced stiffness in the Achilles tendon. These structural differences may reflect adaptive changes in gait patterns associated with aging. Under dual-task conditions, cognitive load appeared to further amplify the age-related modulation of these structure–function relationships. Specific mechanical properties may serve as indicators of gait changes in certain contexts, but their clinical utility requires further validation.

## Figures and Tables

**Table 1:** Subject characteristics.

Parameters		Older Adults	Younger Adults
Age (years)		72.05 ± 3.52	24.8 ± 2.36 **
Sex	Male	8 (36%)	13 (50%)
	Female	14 (64%)	13 (50%)
Height (cm)		159.95 ± 6.55	168.47 ± 8.89 **
Weight (kg)		63.48 ± 10.67	61.70 ± 11.81
BMI (kg/m^2^)		24.47 ± 2.86	21.38 ± 2.50 **
SP speed (m/s)		0.822 ± 0.16	1.178± 0.16 **
Fast speed (m/s)		1.025 ± 0.16	1.44 ± 0.15 **
SP Step (cm)		47.06 ± 1.60	63.35 ± 1.47 **
SP + DT Step (cm)		46.61 ± 1.39	63.44 ± 1.28 **
Fast Step (cm)		53.00 ± 1.44	71.67 ± 1.32 **
Fast + DT Step (cm)		53.12 ± 1.36	71.74 ± 1.25 **

Values are presented as mean ± standard deviation. SP = self-paced walking; DT = dual task. ** *p* < 0.001 indicates significant differences between groups (older vs. younger adults).

**Table 2:** Gait variability outcomes under each walking condition for both groups, along with post hoc comparisons between groups.

Group	Step Length Variability (%)			
	SP Speed	SP Speed DT	Fast Speed	Fast Speed
Older	10.26 ± 4.77	9.64 ± 3.40	6.67 ± 1.88	6.87 ± 1.70
Younger	4.87 ± 1.72	4.96 ± 1.97	4.19 ± 0.58	4.19 ± 0.77
Between group comparisons	0.001	0.001	0.001	0.001
**Group**	**Stride Length Variability (%)**			
	**SP Speed**	**SP Speed DT**	**Fast Speed**	**Fast Speed**
Older	7.48 ± 5.89	6.46 ± 3.88	3.58 ± 1.22	3.85 ± 0.97
Younger	2.48 ± 0.72	2.65 ± 1.17	2.19 ± 0.59	2.19 ± 0.47
Between group comparisons	0.001	0.001	0.001	0.001
**Group**	**Gait Cycle Time Variability (%)**			
	**SP Speed**	**SP Speed DT**	**Fast Speed**	**Fast Speed**
Older	8.42 ± 6.76	7.00 ± 5.62	3.09 ± 2.30	3.02 ± 1.25
Younger	1.98 ± 0.64	2.18 ± 1.04	1.78 ± 0.57	1.81 ± 0.34
Between group comparisons	0.001	0.001	0.005	0.001

Group means (±standard deviation) of gait variability parameters across four walking conditions in older and younger adults. SV = step length variability; SLV = stride length variability; GCV = gait cycle time variability; SP = self-paced walking; DT = dual task. Significant between-group differences were observed under all walking conditions (*p* < 0.05).

**Table 3:** Comparison of stiffness (S) and elasticity (D) values of the right lower limb muscles and tendons between older and younger adults.

Parameters		Right Lower Limb		
		Older Adults	Younger Adults	*p*-Value
Stiffness (S)	Rectus femoris	270 (232–317)	251 (197–296)	0.009 *
	Biceps femoris	276 (238–320)	249 (173–319)	0.011 *
	Tibialis anterior	368 (264–469)	400 (327–531)	0.009 *
	Soleus	288 (236–347)	285 (222–339)	0.653
	Patellar tendon	724 (568–871)	585 (456–729)	<0.001 *
	Achilles tendon	725 (593–867)	759 (590–876)	0.047 *
Elasticity (D)	Rectus femoris	2.02 (1.6–2.6)	1.5 (1.3–2.1)	<0.001 *
	Biceps femoris	1.97 (1.7–2.5)	1.3 (0.8–1.7)	<0.001 *
	Tibialis anterior	1.18 (0.8–1.8)	1.1 (0.7–1.4)	0.218
	Soleus	1.76 (1.2–2.7)	1.6 (1.2–2.0)	0.219
	Patellar tendon	0.87 (0.7–1.3)	1.0 (0.8–1.1)	0.006 *
	Achilles tendon	0.82 (0.6–1.7)	0.8 (0.6–1.1)	0.656

S and D values are presented as median (min–max). * *p* < 0.05 indicates significant differences between groups.

**Table 4:** Correlation between gait variability (CV%) and the mechanical properties of muscles and tendons.

Parameters (*n* = 48)		Stiffness (S)						Elasticity (D)					
		RF	BF	TA	SOL	PT	AT	RF	BF	TA	SOL	PT	AT
SV	SP	0.23	0.20	−0.23	0.12	0.64 **	−0.32 *	0.64 **	0.59 **	0.05	0.30 *	−0.31 *	0.03
	SP + DT	0.31 *	0.25	−0.15	0.16	0.63 **	−0.23	0.63 **	0.60 **	0.06	0.27	−0.34 *	−0.01
	Fast	0.37 **	0.28	−0.24	0.09	0.66 **	−0.24	0.67 **	0.67 **	0.05	0.20	−0.38 **	0.03
	Fast + DT	0.33 *	0.25	−0.24	0.13	0.58 **	−0.22	0.61 **	0.65 **	0.17	0.19	−0.23	−0.02
SLV	SP	0.23	0.19	−0.26	0.08	0.66 **	−0.36 *	0.64 **	0.62 **	0.07	0.21	−0.38 **	0.06
	SP + DT	0.11	0.14	−0.12	0.09	0.63 **	−0.34 *	0.55 **	0.58 **	0.04	0.29 *	−0.39 **	0.13
	Fast	0.28	0.28	−0.21	0.04	0.66 **	−0.22	0.62 **	0.66 **	0.07	0.15	−0.33 *	0.11
	Fast + DT	0.19	0.20	−0.25	0.07	0.63 **	−0.26	0.60 **	0.66 **	0.16	0.28	−0.30 *	0.12
GCV	SP	0.23	0.27	−0.18	0.16	0.68 **	−0.32 *	0.60 **	0.69 **	0.10	0.25	−0.37 **	−0.02
	SP + DT	0.15	0.20	−0.19	0.08	0.62 **	−0.40 **	0.56 **	0.62 **	0.22	0.33 *	−0.24	0.19
	Fast	0.30 *	0.24	−0.06	0.01	0.55 **	−0.18	0.51 **	0.56 **	0.17	0.16	−0.30 *	0.02
	Fast + DT	0.17	0.12	−0.03	0.01	0.59 **	−0.37 *	0.49 **	0.58 **	0.19	0.22	−0.21	0.09

SV = step length variability; SLV = stride length variability; GCV = gait cycle time variability. * *p* < 0.05, ** *p* < 0.01. SP = self-paced walking; DT = dual task.

## Data Availability

The datasets utilized and analyzed in this study can be obtained from the corresponding author upon reasonable request.

## Appendix A

**Table A1:** This table presents regression results examining the associations between tendon-muscle mechanical properties and gait variability (step variability, SV) under four walking conditions: SP = self-paced walking; SP + DT = self-paced dual-task; Fast = fast walking; Fast + DT = fast dual-task walking. β: Regression coef-ficient indicating the effect size and direction of each predictor on SV; *p*-value: Significance level for β; β (+Age): Regression coefficient after adjusting for age; *p*-value (+Age): Significance level after adjusting for age; Δβ: Change in the regression coefficient before and after adjusting for age; Adjusted R^2^ (+Age): Model fit (adjusted R-squared) after including age; Age *p*-value: Significance level of the age variable itself in the adjusted regression model, indicating its independent contribution to predicting SV; ΔF: F-statistic of the original model (without age), assessing overall model significance; ΔF (+Age): F-statistic of the age-adjusted model, assessing whether adding age improves model significance.

	Condition	Predictor	*β*	*p-*Value	*β* (+Age)	*p*-Value (+Age)	Δ*β*	Age *p*-Value	Adjusted R^2^	Adjusted R^2^ (+Age)	ΔF	ΔF (+Age)
SV	SP	PT_S	0.583	<0.001	0.262	0.102	0.321	0.006	0.326	0.419	23.706 (<0.001)	8.394 (0.006)
	SP + DT	PT_S	0.607	<0.001	0.235	0.119	0.372	<0.001	0.355	0.487	26.883 (<0.001)	12.831 (<0.001)
	SP	AT_S	−0.191	0.385	−0.037	0.193	0.154	0.753	0.016	0.384	1.746 (0.193)	28.558 (<0.001)
	SP + DT	AT_S	−0.128	0.385	0.044	0.695	0.172	0.695	−0.005	0.46	0.77 (0.385)	40.62 (<0.001)
	SP	RF_D	0.545	<0.001	0.125	0.491	0.42	0.004	0.282	0.39	19.440 (<0.001)	9.125 (0.004)
	SP + DT	RF_D	0.514	<0.001	−0.056	0.741	0.57	<0.001	0.248	0.46	16.514 (<0.001)	18.993 (<0.001)
	SP	BF_D	0.534	<0.001	−0.086	0.711	0.62	0.003	0.269	0.385	18.308 (<0.001)	9.661 (0.003)
	SP + DT	BF_D	0.549	<0.001	−0.212	0.325	0.761	<0.001	0.286	0.47	19.843 (<0.001)	16.941 (<0.001)
SLV	SP	PT_S	0.364	0.011	−0.529	0.599	0.893	<0.001	0.114	0.311	7.019 (0.011)	14.189 (<0.001)
	SP + DT	PT_S	0.553	<0.001	0.271	0.108	0.282	0.019	0.291	0.359	20.277 (<0.001)	5.889 (0.019)
	SP	AT_S	−0.206	0.159	−0.553	0.583	0.347	<0.001	0.022	0.311	2.048 (0.159)	20.349 (<0.001)
	SP + DT	AT_S	−0.203	0.167	−0.503	0.618	0.3	<0.001	0.02	0.325	1.972 (0.167)	21.719 (<0.001)
	SP	RF_D	0.46	0.001	0.029	0.881	0.431	0.006	0.195	0.307	12.354 (0.001)	8.472 (0.006)
	SP + DT	RF_D	0.438	0.002	−0.048	0.802	0.486	0.002	0.175	0.322	10.93 (0.002)	10.987 (0.002)
	SP	BF_D	0.421	0.003	−0.329	0.175	0.75	<0.001	0.16	0.335	9.924 (0.003)	13.118 (<0.001)
	SP + DT	BF_D	0.504	<0.001	−0.034	0.89	0.538	0.014	0.238	0.321	15.7 (<0.001)	6.607 (0.014)
GCV	SP	PT_S	0.512	<0.001	0.214	0.213	0.298	0.017	0.246	0.322	16.321 (<0.001)	6.183 (0.017)
	SP + DT	PT_S	0.363	0.012	−0.082	0.63	0.445	<0.001	0.113	0.318	6.842 (0.012)	14.521 (<0.001)
	SP	AT_S	−0.087	0.558	0.056	0.656	0.143	<0.001	−0.014	0.301	0.349 (0.558)	21.758 (<0.001)
	SP + DT	AT_S	−0.278	0.058	−0.105	0.413	0.173	<0.001	0.057	0.325	3.782 (0.058)	18.820 (<0.001)
	SP	RF_D	0.411	0.004	−0.079	0.685	0.49	0.002	0.151	0.301	9.375 (0.004)	10.834 (0.002)
	SP + DT	RF_D	0.352	0.015	−0.25	0.191	0.602	<0.001	0.104	0.34	6.348 (0.015)	17.126 (<0.001)
	SP	BF_D	0.501	<0.001	0.017	0.946	0.484	0.028	0.235	0.298	15.399 (<0.001)	5.175 (<0.001)
	SP + DT	BF_D	0.434	0.002	−0.284	0.24	0.718	<0.001	0.17	0.336	10.423 (0.002)	12.206 (0.001)
SV	Fast	PT_S	0.582	<0.001	0.155	0.292	0.427	<0.001	0.324	0.501	23.535 (<0.001)	17.266 (<0.001)
	Fast + DT	PT_S	0.589	<0.001	0.145	0.312	0.444	<0.001	0.333	0.525	24.444 (<0.001)	19.664 (<0.001)
	Fast	AT_S	−0.267	0.067	−0.098	0.362	−0.169	<0.001	0.051	0.498	3.519 (0.067)	41.886 (<0.001)
	Fast + DT	AT_S	−0.136	0.356	0.045	0.43	−0.181	<0.001	−0.003	0.516	0.868 (0.356)	50.375 (<0.001)
	Fast	RF_D	0.624	<0.001	0.179	0.277	0.445	<0.001	0.376	0.501	29.363 (<0.001)	12.546 (<0.001)
	Fast + DT	RF_D	0.555	<0.001	−0.026	0.872	0.581	<0.001	0.293	0.515	20.511 (<0.001)	21.970 (<0.001)
	Fast	BF_D	0.67	<0.001	0.204	0.33	0.466	0.013	0.437	0.499	37.427 (<0.001)	6.709 (0.013)
	Fast + DT	BF_D	0.617	<0.001	−0.068	0.741	0.685	<0.001	0.368	0.516	28.346 (<0.001)	15.025 (<0.001)
SLV	Fast	PT_S	0.5	<0.001	0.101	0.535	0.399	<0.001	0.234	0.384	15.321 (<0.001)	12.228 (<0.001)
	Fast + DT	PT_S	0.576	<0.001	0.069	0.609	0.507	<0.001	0.317	0.572	22.828 (<0.001)	28.376 (<0.001)
	Fast	AT_S	−0.252	0.084	−0.102	0.388	−0.15	<0.001	0.043	0.389	3.111 (<0.001)	27.037 (<0.001)
	Fast + DT	AT_S	−0.224	0.126	−0.039	0.693	−0.185	<0.001	0.03	0.571	2.429 (0.126)	59.035 (<0.001)
	Fast	RF_D	0.526	<0.001	0.084	0.647	0.442	0.003	0.261	0.382	17.578 (<0.001)	9.981 (<0.001)
	Fast + DT	RF_D	0.515	<0.001	−0.194	0.198	0.709	<0.001	0.25	0.585	16.625 (<0.001)	38.208 (<0.001)
	Fast	BF_D	0.6	<0.001	0.195	0.399	0.405	0.047	0.346	0.388	25.907 (<0.001)	4.163 (0.047)
	Fast + DT	BF_D	0.595	<0.001	−0.283	0.138	0.878	<0.001	0.339	0.59	25.155 (<0.001)	29.134 (<0.001)
GCV	Fast	PT_S	0.412	0.004	0.211	0.265	0.201	0.134	0.152	0.176	9.422 (0.004)	2.332 (0.134)
	Fast + DT	PT_S	0.531	<0.001	0.354	0.049	0.177	0.157	0.266	0.283	18.027 (<0.001)	2.067 (<0.001)
	Fast	AT_S	−0.003	0.985	0.11	0.429	−0.113	0.002	−0.022	0.164	0.0004 (0.985)	11.241 (0.002)
	Fast + DT	AT_S	0.025	0.863	0.157	0.237	−0.132	<0.001	−0.021	0.241	0.03 (0.863)	16.901 (<0.001)
	Fast	RF_D	0.315	0.029	−0.052	0.806	0.367	0.03	0.08	0.154	5.064 (0.029)	5.026 (0.030)
	Fast + DT	RF_D	0.299	0.039	−0.219	0.281	0.518	0.002	0.07	0.237	4.511 (0.039)	11.114 (0.002)
	Fast	BF_D	0.446	0.002	0.278	0.302	0.168	0.473	0.181	0.173	11.398 (0.002)	0.524 (0.473)
	Fast + DT	BF_D	0.435	0.002	0.003	0.99	0.432	0.061	0.171	0.217	10.716 (0.002)	3.685 (0.061)
